# Temporal trends in antipsychotic prescriptions for pediatric patients using an administrative hospital database in Japan: a retrospective study

**DOI:** 10.1186/s40780-023-00324-8

**Published:** 2024-01-02

**Authors:** Daisuke Kikuchi, Taku Obara, Ryo Obara, Hiroaki Hino, Naoto Suzuki, Shota Kashiwagura, Takashi Watanabe, Ryusuke Ouchi, Kensuke Usui, Kouji Okada

**Affiliations:** 1 Department of Pharmacy, Tohoku Medical and Pharmaceutical University Hospital, 1-12-1 Fukumuro, Miyagino-ku, Sendai, 983-8512 Miyagi Japan; 2 Division of Molecular Epidemiology, Tohoku University Graduate School of Medicine, 2-1, Seiryo-machi, Aoba-ku, Sendai, 980-8573 Miyagi Japan; 3 Division of Preventive Medicine and Epidemiology, Tohoku University Tohoku Medical Megabank Organization, 2-1, Seiryo-machi, Aoba-ku, Sendai, 980-8573 Miyagi Japan; 4 Department of Pharmaceutical Sciences, Tohoku University Hospital, 1-1, Seiryo-machi, Aoba- ku, Sendai, 980-8574 Miyagi Japan; 5 Department of Pharmacy, Kawasaki Kokoro Hospital, 72, Kitakawarayama, Oaza-Kawauchi, Kawasaki-cho, Shibata-gun, Miyagi, 980-8574 Japan; 6 Division of Clinical Pharmaceutics and Pharmacy Practice, Tohoku Medical and Pharmaceutical University, 1-12-1, Fukumuro, Miyagino-ku, Sendai, 980-8512 Miyagi Japan

**Keywords:** Aripiprazole, Pediatrics, Prescription, Prevalence, Schizophrenia

## Abstract

**Background:**

Schizophrenia is a psychiatric disorder characterized by hallucinations, delusions, and other symptoms. Although treatment guidelines for schizophrenia have been established in Japan, drugs are not recommended for pediatric schizophrenia. Additionally, the temporal trends in prescribing antipsychotics for pediatric patients with schizophrenia are unclear. Therefore, we aimed to clarify the trends in antipsychotic prescriptions for Japanese pediatric outpatients from 2015 to 2022.

**Methods:**

Administrative data (as of November 2023) of Japanese pediatric outpatients with schizophrenia aged 0–18 years who visited acute-care diagnosis procedure combination hospitals between January 1, 2015, and December 31, 2022, were included in this study. The target drugs for schizophrenia were all indicated for treating schizophrenia and marketed in Japan as of December 2022. Annual prescription trends for antipsychotics during this period were calculated based on their proportions. The Cochran–Armitage trend test was used to evaluate the proportion of prescriptions for each antipsychotic.

**Results:**

The main drugs prescribed for these patients were aripiprazole and risperidone. Among male patients, the proportion of prescriptions for aripiprazole increased significantly from 21.2% in 2015 to 35.9% in 2022, whereas that for risperidone decreased significantly from 47.9% in 2015 to 36.7% in 2022 (both P < 0.001). Among female patients, the proportion of prescriptions for aripiprazole increased significantly from 21.6% in 2015 to 35.6% in 2022, whereas that for risperidone decreased significantly from 38.6% in 2015 to 24.8% in 2022 (both P < 0.001).

**Conclusions:**

Aripiprazole and risperidone were primarily prescribed for pediatric schizophrenia in Japan during the study period. Additionally, the proportion of aripiprazole prescriptions increased over time.

## Background

Schizophrenia is a psychiatric disorder characterized by hallucinations, delusions, and other symptoms [1], with an estimated lifetime prevalence of 0.48% globally [2] and 0.59% in Japan [3]. Schizophrenia usually emerges in adulthood but can also emerge during childhood. Moreover, the prevalence of childhood schizophrenia is < 1 in 10,000 and significantly increases between the ages of 13 and 18 [4]. According to the children’s psychiatric practitioner textbook, the onset of schizophrenia in children is at age 5, with its prevalence increasing thereafter [5].

However, information on pharmacological treatments for schizophrenia in children versus adults is limited. Some antipsychotics, such as aripiprazole, risperidone, and quetiapine, reportedly reduce positive and negative schizophrenia symptoms in children when compared with placebo in randomized clinical trials (RCTs) [6–8]. The United States Food and Drug Administration (FDA) approved aripiprazole, olanzapine, paliperidone, quetiapine, and risperidone for patients with schizophrenia aged ≥ 13 years [1]. Blonanserin was first approved in 2021 for children with schizophrenia aged ≥ 12 years in Japan [9, 10]. Currently, drugs prescribed for treating pediatric schizophrenia differ between Japan and the United States. Although schizophrenia treatment guidelines have been established in Japan [11], drugs are not recommended for pediatric schizophrenia. Currently, no definitive best practice for the pharmacotherapeutic treatment of pediatric schizophrenia exists in Japan.

Therefore, we aimed to clarify the antipsychotic prescription trends for pediatric patients in Japan using Japanese administrative data.

## Main text

### MDV analyzer and database

This retrospective survey was conducted using the MDV analyzer (Medical Data Vision Co., Ltd., Tokyo, Japan) [12]. We assessed an anonymized administrative hospital database assembled by Medical Data Vision Co., Ltd., containing inpatient and outpatient information from Japanese acute-care hospitals. Data on 45.2 million patients (including deceased patients) from 493 acute-care diagnosis procedure combination (DPC) hospitals were included by the maintenance endpoint in November 2023. The age distributions of these patients were 0–14 (14.2%), 15–64 (50.6%), and > 65 (35.2%) years.

### Study population

Pediatric outpatients with schizophrenia were diagnosed using the International Statistical Classification of Diseases 10th Revision codes for schizophrenia, schizotypal, and delusional disorders (F20–F29). We also investigated the extent of developmental disabilities (F80–F89, F90–99) in patients with schizophrenia. Therefore, the population comprised outpatients with schizophrenia aged 0–18 years who visited acute-care DPC hospitals that provided administrative data to Medical Data Vision Co., Ltd. from January 1, 2015, to December 31, 2022.

### Definition of target antipsychotic prescriptions

The target antipsychotic prescriptions were all indicated for treating schizophrenia and marketed in Japan as of December 2022, irrespective of whether they were indicated for pediatric use in Japan. The prescribed drugs were tabulated annually from 2015 to 2022 to eliminate the effect of variations in prescription within each year and extracted from the Japanese administrative data in the MDV analyzer.

### Evaluation items

The evaluation items included schizophrenia prevalence, the proportion of antipsychotic prescriptions, and each antipsychotic prescribed. Since the patients’ raw data could not be identified in the MDV analyzer, the schizophrenia prevalence and proportion of each prescription were calculated using available data and the following formulas:

Schizophrenia prevalence = (number of patients with schizophrenia/number of all patients) ×100.

Developmental disorders among patients with schizophrenia = (number of patients with developmental disorders in patients with schizophrenia/number of patients with schizophrenia) ×100.

Proportion of antipsychotic prescriptions = (number of patients prescribed antipsychotics/number of patients with schizophrenia) ×100.

Additionally, the prescribing trends for patients with schizophrenia with and without developmental disorders were compared.

### Statistical analysis

The proportion of prescriptions for antipsychotic from 2015 to 2022 were calculated and compared using the Cochran–Armitage trend test. Considering the age categories in previous studies [3, 4], these values were stratified by sex and age (0–4, 5–12, and 13–18 years). Statistical significance was set at P < 0.05. All statistical analyses were performed using R ver. 4.2.3 (R Foundation for Statistical Computing, Vienna, Austria) [13].

## Results

**Fig. 1:**
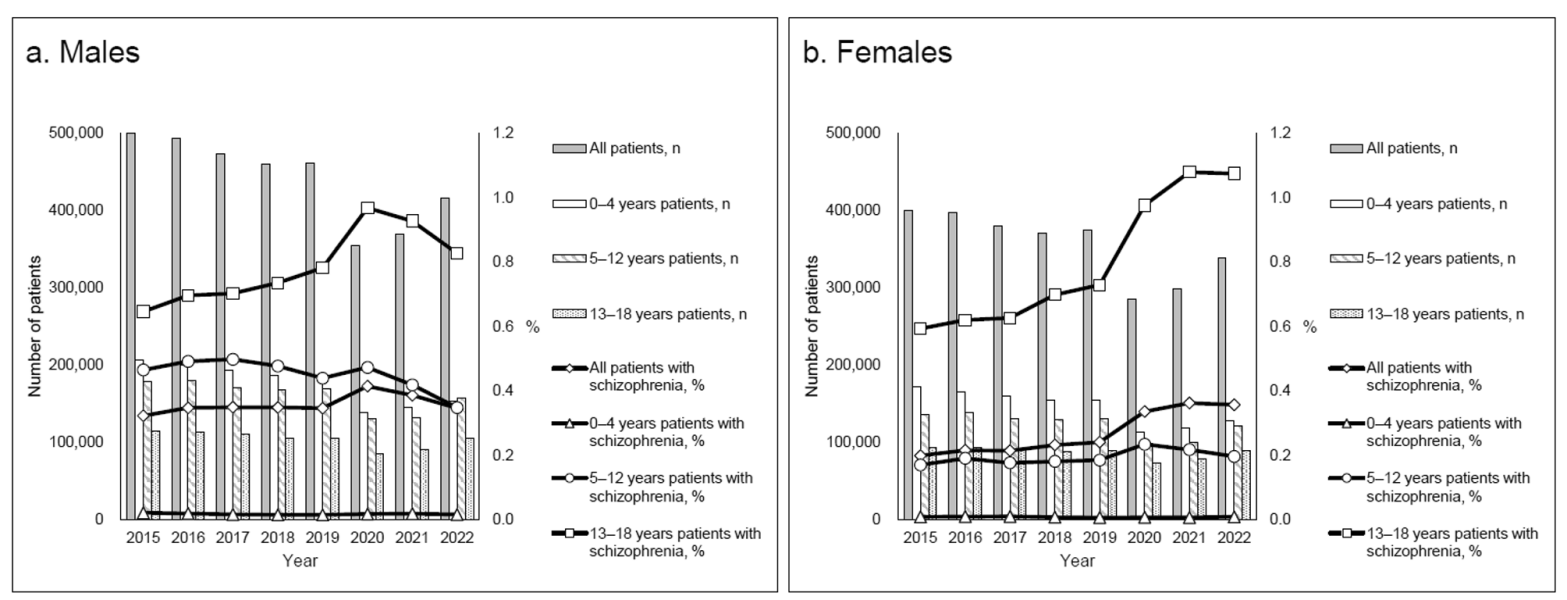
Number of patients enrolled in the MDV analyzer and proportion of schizophrenia prevalence by year. The horizontal, left vertical, and right vertical axes show the year, number of patients enrolled in the MDV analyzer, and proportion of schizophrenia prevalence (%), respectively

Schizophrenia prevalence increased with age (Fig. 1). Table 1 presents the yearly proportions of antipsychotic prescriptions for schizophrenia. Aripiprazole and risperidone were the most prescribed drugs. Among males, the proportion of prescriptions for aripiprazole increased significantly from 21.2% in 2015 to 35.9% in 2022, whereas that for risperidone decreased significantly from 47.9% in 2015 to 36.7% in 2022 (both P < 0.001). Among females, the proportion of prescriptions for aripiprazole increased significantly from 21.6% in 2015 to 35.6% in 2022, whereas that for risperidone decreased significantly from 38.6% in 2015 to 24.8% in 2022 (both P < 0.001). The proportion of prescriptions for blonanserin was 0.3% and 1.3% for males and females in 2022, respectively.

**Table 1:** Proportions of antipsychotic prescriptions by year (including patients with developmental disabilities)

	2015	2016	2017	2018	2019	2020	2021	2022	*P*
Males									
All patients									
Patients with schizophrenia, n	1612	1712	1650	1602	1595	1469	1425	1441	
Prescriptions for any antipsychotic, %	78.3	78.5	75.6	76.7	75.3	73.3	73.1	76.9	< 0.001
Risperidone, %	47.9	46.5	44.8	43.4	41.4	42.2	38.7	36.7	< 0.001
Aripiprazole, %	21.2	26.3	27.2	29.5	29.7	29.0	30.8	35.9	< 0.001
Quetiapine, %	5.3	4.7	5.0	4.3	4.8	5.0	6.3	6.2	0.052
Olanzapine, %	3.9	3.2	3.2	2.6	2.4	2.8	4.4	5.0	0.07
Haloperidol, %	4.0	3.1	3.0	2.4	1.9	2.3	2.0	2.1	< 0.001
Sulpiride, %	1.0	0.8	1.0	0.9	0.8	1.0	1.6	1.9	0.002
Levomepromazine, %	2.2	1.6	1.6	1.3	1.2	1.5	1.8	1.9	0.64
Brexpiprazole, %	0.0	0.0	0.0	0.1	0.9	0.9	0.8	1.0	< 0.001
Lurasidone, %	0.0	0.0	0.0	0.0	0.0	0.1	0.6	1.0	< 0.001
Perospirone, %	0.8	0.6	0.5	0.2	0.6	0.9	0.6	0.9	0.45
Chlorpromazine, %	0.9	1.1	0.8	0.8	0.8	0.7	0.7	0.8	0.20
Propericiazine, %	0.4	0.4	0.4	0.2	0.3	0.5	0.7	0.6	0.07
Zotepine, %	0.2	0.2	0.3	0.1	0.1	0.2	0.1	0.3	0.92
Paliperidone, %	0.7	0.6	0.4	0.4	0.5	0.3	0.3	0.3	0.05
Blonanserin, %	0.7	0.3	0.2	0.3	0.5	0.6	0.6	0.3	0.85
Asenapine, %	0.0	0.1	0.1	0.2	0.1	0.4	0.3	0.1	0.02
Prochlorperazine, %	0.1	0.2	0.0	0.1	0.5	0.1	0.1	0.1	0.81
Clozapine, %	0.0	0.1	0.1	0.1	0.2	0.1	0.0	0.0	0.96
Sultopride, %	0.0	0.0	0.1	0.0	0.0	0.0	0.0	0.0	0.55
Fluphenazine, %	0.0	0.1	0.0	0.0	0.0	0.1	0.0	0.0	0.82
Bromperidol, %	0.1	0.1	0.1	0.1	0.0	0.1	0.0	0.0	0.25
0–4 years									
Patients with schizophrenia, n	44	38	31	28	28	24	27	25	
Prescriptions for any antipsychotic, %	84.1	73.7	61.3	75.0	64.3	75.0	66.7	60.0	0.06
Aripiprazole, %	15.9	18.4	25.8	28.6	21.4	25.0	25.9	32.0	0.12
Risperidone, %	70.5	55.3	38.7	50.0	46.4	58.3	40.7	32.0	0.01
Chlorpromazine, %	0.0	2.6	0.0	0.0	0.0	0.0	0.0	4.0	0.57
Quetiapine, %	2.3	2.6	3.2	7.1	3.6	0.0	0.0	0.0	0.34
Haloperidol, %	0.0	2.6	0.0	0.0	0.0	4.2	3.7	0.0	0.49
Propericiazine, %	0.0	0.0	3.2	0.0	0.0	0.0	0.0	0.0	0.65
Levomepromazine, %	2.3	0.0	0.0	0.0	0.0	0.0	0.0	0.0	0.19
5–12 years									
Patients with schizophrenia, n	831	887	847	799	744	615	553	549	
Prescriptions for any antipsychotic, %	80.0	78.4	77.8	77.6	74.5	74.1	73.2	79.6	0.02
Aripiprazole, %	23.3	30.0	31.1	34.4	35.5	34.8	36.2	43.4	< 0.001
Risperidone, %	55.6	51.6	50.9	48.6	43.8	46.2	44.7	42.8	< 0.001
Quetiapine, %	2.8	2.5	2.0	1.6	3.1	2.4	2.4	3.6	0.36
Haloperidol, %	4.1	3.0	3.0	2.4	1.6	2.6	2.2	2.2	0.01
Olanzapine, %	0.7	0.2	0.5	0.4	0.3	0.7	1.1	1.8	0.01
Levomepromazine, %	1.1	0.9	1.2	0.8	0.7	0.5	0.2	0.7	0.06
Chlorpromazine, %	0.4	0.7	0.5	0.4	0.3	0.0	0.2	0.4	0.15
Sulpiride, %	0.5	0.3	0.2	0.5	0.4	0.3	0.9	0.4	0.53
Propericiazine, %	0.6	0.3	0.4	0.3	0.3	0.3	0.0	0.4	0.20
Perospirone, %	0.8	0.6	0.2	0.1	0.1	0.3	0.4	0.4	0.11
Lurasidone, %	0.0	0.0	0.0	0.0	0.0	0.0	0.0	0.4	0.01
Paliperidone, %	0.1	0.1	0.0	0.1	0.1	0.0	0.2	0.2	0.68
Blonanserin, %	0.1	0.0	0.0	0.1	0.0	0.2	0.2	0.2	0.28
Brexpiprazole, %	0.0	0.0	0.0	0.0	0.0	0.2	0.0	0.0	0.40
Prochlorperazine, %	0.0	0.0	0.0	0.0	0.1	0.0	0.0	0.0	0.69
13–18 years									
Patients with schizophrenia, n	737	787	772	775	823	830	845	867	
Prescriptions for any antipsychotic, %	76.1	78.9	73.8	75.9	76.4	72.7	73.1	75.7	0.08
Risperidone, %	43.0	46.9	43.3	43.9	43.4	42.8	38.2	37.6	< 0.001
Aripiprazole, %	21.2	25.0	24.4	27.9	28.9	28.0	30.8	35.5	< 0.001
Quetiapine, %	9.0	7.6	8.7	7.2	7.0	7.3	9.5	8.3	0.91
Olanzapine, %	8.0	6.9	6.3	5.2	4.4	4.6	6.7	7.4	0.40
Sulpiride, %	1.6	1.4	1.8	1.3	1.2	1.6	2.2	3.1	0.02
Levomepromazine, %	3.7	2.7	2.5	2.2	1.7	2.5	3.0	2.8	0.55
Haloperidol, %	4.5	3.4	3.4	2.6	2.3	2.3	2.2	2.4	0.002
Brexpiprazole, %	0.0	0.0	0.0	0.1	1.7	1.4	1.3	1.6	< 0.001
Lurasidone, %	0.0	0.0	0.0	0.0	0.0	0.1	0.9	1.4	< 0.001
Perospirone, %	1.1	0.8	0.9	0.4	1.1	1.3	0.8	1.3	0.40
Chlorpromazine, %	1.8	1.8	1.3	1.3	1.3	1.2	1.1	0.9	0.06
Propericiazine, %	0.1	0.4	0.4	0.1	0.5	0.8	1.2	0.8	0.002
Zotepine, %	0.4	0.4	0.6	0.3	0.2	0.4	0.2	0.5	0.68
Paliperidone, %	1.4	1.1	0.8	0.8	1.0	0.6	0.4	0.5	0.01
Blonanserin, %	1.5	0.6	0.4	0.5	1.0	1.0	0.8	0.3	0.24
Asenapine, %	0.0	0.1	0.1	0.4	0.2	0.7	0.5	0.2	0.052
Prochlorperazine, %	0.3	0.5	0.0	0.3	0.9	0.2	0.1	0.1	0.45
Clozapine, %	0.0	0.1	0.3	0.1	0.4	0.2	0.0	0.0	0.72
Sultopride, %	0.0	0.0	0.1	0.0	0.0	0.0	0.0	0.0	0.48
Fluphenazine, %	0.0	0.1	0.0	0.0	0.0	0.1	0.0	0.0	0.71
Bromperidol, %	0.1	0.1	0.1	0.1	0.0	0.1	0.0	0.0	0.17
Females									
All patients									
Patients with schizophrenia, n	796	854	814	860	900	956	1079	1208	
Prescriptions for any antipsychotic, %	77.1	75.9	72.2	75.5	73.9	73.8	76.9	76.4	0.67
Aripiprazole, %	21.6	24.9	27.4	28.7	30.9	29.8	34.4	35.6	< 0.001
Risperidone, %	38.6	38.1	34.3	34.3	32.2	32.4	28.2	24.8	< 0.001
Quetiapine, %	16.3	14.2	14.0	16.0	16.0	16.5	19.5	18.1	0.002
Olanzapine, %	7.2	7.8	7.5	6.7	6.6	5.6	8.5	8.3	0.46
Sulpiride, %	2.6	2.0	2.0	1.9	2.6	2.5	3.6	4.6	< 0.001
Lurasidone, %	0.0	0.0	0.0	0.0	0.0	0.4	3.0	4.3	< 0.001
Brexpiprazole, %	0.0	0.0	0.0	0.6	2.6	3.3	3.2	3.1	< 0.001
Levomepromazine, %	1.5	2.5	2.6	3.7	3.1	1.8	2.2	2.3	0.98
Perospirone, %	3.0	2.2	2.3	1.5	1.8	2.0	2.4	2.2	0.44
Chlorpromazine, %	1.8	1.2	1.7	1.9	1.8	1.5	1.7	2.0	0.51
Blonanserin, %	1.4	1.9	2.3	1.9	2.0	1.4	1.5	1.3	0.28
Haloperidol, %	2.9	3.5	2.6	2.1	2.4	2.7	1.9	0.8	< 0.001
Asenapine, %	0.0	0.2	0.5	0.5	0.6	0.7	0.3	0.5	0.14
Paliperidone, %	0.9	0.9	0.5	0.3	0.0	0.5	0.5	0.4	0.07
Prochlorperazine, %	0.3	0.0	0.0	0.2	0.4	0.1	0.3	0.2	0.50
Zotepine, %	0.6	0.4	0.1	0.3	0.3	0.3	0.2	0.1	0.06
Pipamperone, %	0.0	0.0	0.0	0.0	0.0	0.1	0.0	0.1	0.18
Propericiazine, %	0.6	0.8	0.5	0.2	0.2	0.2	0.2	0.1	0.001
Oxypertine, %	0.1	0.0	0.0	0.0	0.0	0.0	0.0	0.0	0.10
Clozapine, %	0.0	0.2	0.1	0.1	0.0	0.0	0.0	0.0	0.08
Fluphenazine, %	0.1	0.1	0.1	0.0	0.0	0.0	0.0	0.0	0.04
Bromperidol, %	0.1	0.0	0.0	0.0	0.0	0.0	0.0	0.0	0.10
0–4 years									
Patients with schizophrenia, n	15	16	16	12	10	8	8	11	
Prescriptions for any antipsychotic, %	73.3	81.3	62.5	58.3	60.0	50.0	50.0	63.6	0.15
Risperidone, %	46.7	50.0	43.8	41.7	40.0	37.5	25.0	54.5	0.69
Aripiprazole, %	26.7	18.8	25.0	33.3	50.0	12.5	0.0	18.2	0.45
Quetiapine, %	0.0	6.3	12.5	16.7	20.0	12.5	12.5	0.0	0.66
Sulpiride, %	0.0	6.3	0.0	0.0	0.0	0.0	0.0	0.0	0.38
Haloperidol, %	0.0	0.0	0.0	0.0	0.0	0.0	12.5	0.0	0.19
5–12 years									
Patients with schizophrenia, n	231	264	230	233	242	231	218	239	
Prescriptions for any antipsychotic, %	79.7	74.6	73.0	75.1	75.2	68.8	77.1	81.2	0.75
Aripiprazole, %	24.2	27.7	28.3	24.5	28.1	25.1	35.3	39.7	< 0.001
Risperidone, %	50.6	45.1	43.9	49.8	45.5	45.9	39.4	36.8	0.004
Quetiapine, %	9.1	6.4	5.2	6.9	6.2	3.9	8.7	9.2	0.72
Olanzapine, %	0.9	1.1	0.4	0.9	2.5	3.0	1.8	4.2	0.001
Sulpiride, %	1.3	1.5	0.4	0.0	0.8	0.4	1.4	2.1	0.53
Chlorpromazine, %	0.9	0.8	0.4	0.4	0.0	0.4	0.5	1.3	0.90
Haloperidol, %	4.3	3.8	3.0	1.7	3.7	3.9	4.6	1.3	0.36
Brexpiprazole, %	0.0	0.0	0.0	0.0	0.4	0.4	1.8	1.3	< 0.001
Blonanserin, %	0.0	0.0	0.4	0.4	0.0	0.4	1.4	0.8	0.03
Perospirone, %	0.0	0.0	0.0	0.0	0.4	1.3	0.5	0.8	0.01
Lurasidone, %	0.0	0.0	0.0	0.0	0.0	0.0	0.0	0.8	0.03
Levomepromazine, %	0.4	0.8	0.9	2.1	2.1	0.9	0.9	0.8	0.67
Paliperidone, %	0.0	0.0	0.0	0.0	0.0	0.4	0.9	0.4	0.03
Fluphenazine, %	0.0	0.4	0.0	0.0	0.0	0.0	0.0	0.0	0.28
Prochlorperazine, %	0.0	0.0	0.0	0.0	0.0	0.0	0.5	0.0	0.27
Propericiazine, %	0.9	0.8	0.0	0.0	0.0	0.0	0.0	0.0	0.01
13–18 years									
Patients with schizophrenia, n	550	574	568	615	648	717	853	958	
Prescriptions for any antipsychotic, %	76.2	76.3	72.2	75.9	73.6	75.7	77.1	75.4	0.67
Aripiprazole, %	22.0	25.4	27.8	31.1	32.6	32.8	35.5	35.3	< 0.001
Risperidone, %	34.9	36.9	31.5	30.4	28.4	30.0	26.1	23.0	< 0.001
Quetiapine, %	20.0	18.3	18.0	19.7	20.1	21.2	22.5	20.7	0.07
Olanzapine, %	10.0	11.3	10.6	9.3	8.2	6.7	10.3	9.7	0.29
Sulpiride, %	3.3	2.3	2.6	2.6	3.2	3.2	4.2	5.4	< 0.001
Lurasidone, %	0.0	0.0	0.0	0.0	0.0	0.6	3.8	5.3	< 0.001
Brexpiprazole, %	0.0	0.0	0.0	0.8	3.5	4.3	3.6	3.7	< 0.001
Perospirone, %	4.4	3.3	3.3	2.1	2.3	2.2	2.9	2.7	0.09
Levomepromazine, %	2.0	3.3	3.3	4.6	3.5	2.1	2.7	2.7	0.57
Chlorpromazine, %	2.2	1.6	2.3	2.6	2.5	1.8	2.0	2.2	0.97
Blonanserin, %	2.0	2.8	3.2	2.4	2.8	1.7	1.5	1.5	0.03
Haloperidol, %	2.5	3.5	2.6	2.3	2.3	2.4	1.2	0.8	< 0.001
Asenapine, %	0.0	0.3	0.7	0.7	0.8	1.0	0.4	0.6	0.24
Paliperidone, %	1.3	1.4	0.7	0.5	0.0	0.6	0.6	0.4	0.01
Prochlorperazine, %	0.4	0.0	0.0	0.3	0.6	0.1	0.2	0.2	0.82
Zotepine, %	0.9	0.5	0.2	0.5	0.5	0.4	0.2	0.1	0.03
Pipamperone, %	0.0	0.0	0.0	0.0	0.0	0.1	0.0	0.1	0.21
Propericiazine, %	0.7	0.9	0.7	0.3	0.3	0.3	0.2	0.1	0.01
Oxypertine, %	0.2	0.0	0.0	0.0	0.0	0.0	0.0	0.0	0.09
Clozapine, %	0.0	0.3	0.2	0.2	0.0	0.0	0.0	0.0	0.06
Fluphenazine, %	0.2	0.2	0.2	0.0	0.0	0.0	0.0	0.0	0.03
Bromperidol, %	0.2	0.0	0.0	0.0	0.0	0.0	0.0	0.0	0.09

Each drug is listed in the order of the proportion of prescriptions for antipsychotics in 2022

The Cochran–Armitage trend test was used for statistical analysis

*P* < 0.05 was considered statistically significant

**Fig. 2:**
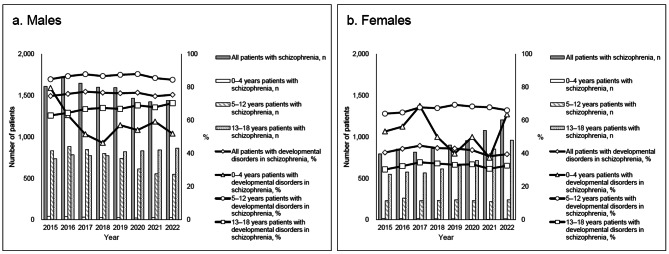
Proportions of developmental disorders among patients with schizophrenia by year. The horizontal, left vertical, and right vertical axes show the year, number of schizophrenia cases, and proportion of developmental disorders in schizophrenia patients (%), respectively

Furthermore, Fig. 2 presents the prevalence of developmental disorders in patients with schizophrenia. Table 2 presents the proportions of antipsychotic prescriptions for schizophrenic patients without developmental disorders. Aripiprazole and risperidone showed similar trends in schizophrenic patients with developmental disabilities.

**Table 2:** Proportions of antipsychotic prescriptions by year (excluding patients with developmental disabilities)

	2015	2016	2017	2018	2019	2020	2021	2022	*P*
Males									
All patients									
Patients with schizophrenia, n	408	411	374	373	377	342	362	354	
Prescriptions for any antipsychotic, %	78.4	79.8	78.3	75.1	73.5	77.5	71.8	77.4	0.06
Aripiprazole, %	18.6	20.2	21.7	22.5	25.7	28.1	28.7	30.8	< 0.001
Risperidone, %	41.2	44.5	40.6	40.2	33.7	38.6	31.5	29.7	< 0.001
Quetiapine, %	9.3	8.3	10.2	7.8	9.5	11.1	11.3	11.9	0.06
Olanzapine, %	9.8	8.3	10.2	7.2	6.9	6.4	8.6	10.5	0.73
Sulpiride, %	2.0	1.5	1.9	1.1	1.3	1.8	3.3	5.6	< 0.001
Brexpiprazole, %	0.0	0.0	0.0	0.3	3.4	3.2	1.9	2.8	< 0.001
Lurasidone, %	0.0	0.0	0.0	0.0	0.0	0.3	1.4	2.8	< 0.001
Perospirone, %	1.5	1.2	1.6	0.5	0.8	1.8	0.8	2.3	0.62
Haloperidol, %	4.7	2.9	1.9	2.1	1.6	1.5	1.9	1.7	0.005
Chlorpromazine, %	1.7	1.0	0.8	1.6	1.1	1.2	1.4	1.4	0.97
Levomepromazine, %	2.0	2.2	2.4	1.6	0.8	2.0	1.9	0.8	0.21
Blonanserin, %	1.7	1.0	0.5	0.8	0.8	1.5	1.4	0.6	0.59
Propericiazine, %	0.2	0.0	0.8	0.3	0.8	0.3	0.6	0.6	0.33
Paliperidone, %	1.7	1.7	0.8	1.1	1.1	0.6	0.6	0.3	0.01
Prochlorperazine, %	0.5	1.0	0.0	0.5	2.1	0.6	0.3	0.3	0.80
Asenapine, %	0.0	0.2	0.3	0.8	0.3	1.5	0.3	0.0	0.42
Clozapine, %	0.0	0.2	0.5	0.3	0.8	0.6	0.0	0.0	0.95
Zotepine, %	0.5	0.7	0.8	0.0	0.0	0.0	0.0	0.0	0.01
Fluphenazine, %	0.0	0.2	0.0	0.0	0.0	0.3	0.0	0.0	0.82
Bromperidol, %	0.2	0.2	0.0	0.0	0.0	0.3	0.0	0.0	0.30
0–4 years									
Patients with schizophrenia, n	9	14	15	15	12	11	11	12	
Prescriptions for any antipsychotic, %	66.7	57.1	53.3	66.7	50.0	72.7	63.6	50.0	0.87
Risperidone, %	66.7	42.9	40.0	60.0	41.7	54.5	36.4	25.0	0.16
Aripiprazole, %	11.1	7.1	6.7	6.7	16.7	27.3	27.3	16.7	0.10
Chlorpromazine, %	0.0	0.0	0.0	0.0	0.0	0.0	0.0	8.3	0.11
Quetiapine, %	0.0	0.0	0.0	6.7	0.0	0.0	0.0	0.0	0.84
Haloperidol, %	0.0	7.1	0.0	0.0	0.0	0.0	0.0	0.0	0.26
Propericiazine, %	0.0	0.0	6.7	0.0	0.0	0.0	0.0	0.0	0.51
5–12 years									
Patients with schizophrenia, n	126	118	103	106	93	74	80	85	
Prescriptions for any antipsychotic, %	89.7	83.9	82.5	77.4	75.3	75.7	73.8	83.5	0.01
Aripiprazole, %	17.5	19.5	21.4	21.7	24.7	31.1	32.5	43.5	< 0.001
Risperidone, %	63.5	63.6	61.2	56.6	41.9	39.2	41.3	40.0	< 0.001
Quetiapine, %	5.6	3.4	2.9	1.9	9.7	6.8	5.0	7.1	0.21
Olanzapine, %	2.4	0.0	1.9	0.9	2.2	1.4	1.3	2.4	0.76
Chlorpromazine, %	0.8	0.8	1.0	0.9	0.0	0.0	1.3	1.2	0.97
Sulpiride, %	0.0	0.0	0.0	0.0	1.1	1.4	2.5	1.2	0.02
Paliperidone, %	0.0	0.0	0.0	0.0	0.0	0.0	1.3	1.2	0.04
Haloperidol, %	4.8	3.4	1.9	1.9	2.2	4.1	2.5	1.2	0.23
Blonanserin, %	0.0	0.0	0.0	0.9	0.0	1.4	1.3	1.2	0.07
Perospirone, %	1.6	1.7	0.0	0.0	0.0	0.0	0.0	1.2	0.19
Lurasidone, %	0.0	0.0	0.0	0.0	0.0	0.0	0.0	1.2	0.09
Prochlorperazine, %	0.0	0.0	0.0	0.0	1.1	0.0	0.0	0.0	0.71
Propericiazine, %	0.8	0.0	1.0	0.0	0.0	0.0	0.0	0.0	0.19
Levomepromazine, %	1.6	0.8	2.9	0.9	0.0	0.0	0.0	0.0	0.05
13–18 years									
Patients with schizophrenia, n	273	279	256	252	272	257	271	257	
Prescriptions for any antipsychotic, %	73.6	79.2	78.1	74.6	73.9	78.2	71.6	76.7	0.59
Aripiprazole, %	20.1	22.2	22.7	25.0	26.5	28.8	28.0	30.0	< 0.001
Risperidone, %	33.3	40.5	35.2	34.9	32.4	38.5	29.5	29.2	0.04
Quetiapine, %	12.1	11.1	13.7	10.7	10.7	13.2	13.7	14.4	0.30
Olanzapine, %	13.9	12.2	14.1	10.7	8.8	8.2	11.1	13.6	0.27
Sulpiride, %	2.9	2.2	2.7	1.6	1.5	1.9	3.7	7.8	0.00
Brexpiprazole, %	0.0	0.0	0.0	0.4	4.8	4.3	2.6	3.9	< 0.001
Lurasidone, %	0.0	0.0	0.0	0.0	0.0	0.4	1.8	3.5	< 0.001
Perospirone, %	1.5	1.1	2.3	0.8	1.1	2.3	1.1	2.7	0.37
Haloperidol, %	4.8	2.5	2.0	2.4	1.5	0.8	1.8	1.9	0.02
Chlorpromazine, %	2.2	1.1	0.8	2.0	1.5	1.6	1.5	1.2	0.72
Levomepromazine, %	2.2	2.9	2.7	2.0	1.1	2.7	2.6	1.2	0.41
Propericiazine, %	0.0	0.0	0.4	0.4	1.1	0.4	0.7	0.8	0.07
Blonanserin, %	2.6	1.4	0.8	0.8	1.1	1.6	1.5	0.4	0.16
Prochlorperazine, %	0.7	1.4	0.0	0.8	2.6	0.8	0.4	0.4	0.62
Paliperidone, %	2.6	2.5	1.2	1.6	1.5	0.8	0.4	0.0	< 0.001
Zotepine, %	0.7	1.1	1.2	0.0	0.0	0.0	0.0	0.0	0.00
Bromperidol, %	0.4	0.4	0.0	0.0	0.0	0.4	0.0	0.0	0.27
Asenapine, %	0.0	0.4	0.4	1.2	0.4	1.9	0.4	0.0	0.50
Clozapine, %	0.0	0.4	0.8	0.4	1.1	0.8	0.0	0.0	0.86
Fluphenazine, %	0.0	0.4	0.0	0.0	0.0	0.4	0.0	0.0	0.77
Females									
All patients									
Patients with schizophrenia, n	473	488	450	488	513	554	665	730	
Prescriptions for any antipsychotic, %	76.1	75.6	70.9	78.3	74.1	73.3	79.5	78.8	0.045
Aripiprazole, %	20.7	23.6	24.7	28.5	29.0	30.1	34.6	36.7	< 0.001
Quetiapine, %	22.2	19.9	18.4	22.1	23.0	23.6	25.3	23.6	0.02
Risperidone, %	31.5	32.4	29.3	27.9	26.1	24.2	23.8	20.0	< 0.001
Olanzapine, %	10.4	11.5	10.0	9.6	9.6	7.0	10.7	10.1	0.49
Lurasidone, %	0.0	0.0	0.0	0.0	0.0	0.7	4.1	6.3	< 0.001
Sulpiride, %	3.4	2.0	2.4	2.7	3.3	3.4	5.4	6.2	< 0.001
Brexpiprazole, %	0.0	0.0	0.0	1.0	4.3	5.2	4.2	4.7	< 0.001
Levomepromazine, %	1.5	3.1	2.7	4.3	3.7	2.2	2.3	2.7	0.92
Perospirone, %	4.2	3.3	4.2	2.5	2.3	2.7	3.2	2.6	0.10
Chlorpromazine, %	2.1	1.2	2.0	2.7	2.5	1.8	2.4	2.3	0.41
Blonanserin, %	2.3	2.9	3.8	2.7	1.9	1.6	2.0	1.5	0.03
Asenapine, %	0.0	0.4	0.9	0.6	0.8	0.9	0.3	0.7	0.35
Paliperidone, %	0.8	1.2	0.4	0.4	0.0	0.7	0.5	0.4	0.13
Haloperidol, %	2.1	2.7	2.0	1.8	1.8	1.4	0.8	0.3	< 0.001
Prochlorperazine, %	0.4	0.0	0.0	0.4	0.8	0.2	0.5	0.3	0.56
Zotepine, %	1.1	0.4	0.2	0.6	0.6	0.2	0.2	0.1	0.02
Pipamperone, %	0.0	0.0	0.0	0.0	0.0	0.2	0.0	0.1	0.19
Propericiazine, %	0.4	0.8	0.2	0.0	0.0	0.0	0.3	0.1	0.07
Oxypertine, %	0.2	0.0	0.0	0.0	0.0	0.0	0.0	0.0	0.10
Clozapine, %	0.0	0.4	0.2	0.2	0.0	0.0	0.0	0.0	0.07
Fluphenazine, %	0.2	0.2	0.2	0.0	0.0	0.0	0.0	0.0	0.04
Bromperidol, %	0.2	0.0	0.0	0.0	0.0	0.0	0.0	0.0	0.10
0–4 years									
Patients with schizophrenia, n	7	7	5	6	6	4	5	4	
Prescriptions for any antipsychotic, %	71.4	71.4	80.0	50.0	66.7	25.0	40.0	25.0	0.03
Aripiprazole, %	14.3	14.3	20.0	33.3	50.0	25.0	0.0	25.0	0.77
Quetiapine, %	0.0	0.0	40.0	0.0	16.7	25.0	20.0	0.0	0.47
Haloperidol, %	0.0	0.0	0.0	0.0	0.0	0.0	20.0	0.0	0.20
Risperidone, %	57.1	57.1	40.0	50.0	50.0	0.0	0.0	0.0	0.004
5–12 years									
Patients with schizophrenia, n	83	93	74	76	74	73	70	81	
Prescriptions for any antipsychotic, %	71.0	81.0	53.0	65.0	59.0	46.0	60.0	70.0	0.50
Aripiprazole, %	25.3	30.1	25.7	23.7	31.1	27.4	41.4	45.7	0.002
Risperidone, %	48.2	47.3	44.6	51.3	37.8	30.1	31.4	30.9	< 0.001
Quetiapine, %	16.9	10.8	8.1	15.8	14.9	9.6	18.6	9.9	0.85
Olanzapine, %	2.4	3.2	1.4	2.6	6.8	4.1	4.3	7.4	0.06
Sulpiride, %	1.2	1.1	1.4	0.0	2.7	0.0	2.9	4.9	0.07
Brexpiprazole, %	0.0	0.0	0.0	0.0	1.4	1.4	2.9	3.7	0.003
Chlorpromazine, %	1.2	1.1	0.0	0.0	0.0	1.4	1.4	2.5	0.32
Lurasidone, %	0.0	0.0	0.0	0.0	0.0	0.0	0.0	2.5	0.03
Levomepromazine, %	0.0	1.1	1.4	3.9	5.4	1.4	0.0	2.5	0.43
Paliperidone, %	0.0	0.0	0.0	0.0	0.0	0.0	0.0	1.2	0.12
Blonanserin, %	0.0	0.0	1.4	1.3	0.0	1.4	1.4	1.2	0.24
Haloperidol, %	2.4	3.2	0.0	0.0	1.4	0.0	1.4	0.0	0.08
Fluphenazine, %	0.0	1.1	0.0	0.0	0.0	0.0	0.0	0.0	0.30
Prochlorperazine, %	0.0	0.0	0.0	0.0	0.0	0.0	1.4	0.0	0.26
Propericiazine, %	0.0	1.1	0.0	0.0	0.0	0.0	0.0	0.0	0.30
Perospirone, %	0.0	0.0	0.0	0.0	1.4	2.7	0.0	0.0	0.34
13–18 years									
Patients with schizophrenia, n	383	388	371	406	433	477	590	645	
Prescriptions for any antipsychotic, %	74.2	72.9	70.6	77.3	73.2	75.3	79.2	78.1	0.005
Aripiprazole, %	21.1	22.9	24.5	29.6	28.6	31.9	34.9	35.7	< 0.001
Quetiapine, %	23.8	22.7	20.8	23.9	24.9	26.6	26.3	25.4	0.07
Risperidone, %	28.2	30.2	26.7	24.6	24.2	24.7	23.2	19.5	< 0.001
Olanzapine, %	12.3	13.9	11.9	11.3	10.2	7.5	11.5	10.9	0.10
Lurasidone, %	0.0	0.0	0.0	0.0	0.0	0.8	4.6	7.0	< 0.001
Sulpiride, %	3.9	2.3	2.7	3.2	3.5	4.0	5.8	6.5	< 0.001
Brexpiprazole, %	0.0	0.0	0.0	1.2	5.1	5.9	4.4	4.8	< 0.001
Perospirone, %	5.2	4.1	5.1	3.0	2.5	2.7	3.6	2.9	0.03
Levomepromazine, %	1.8	3.6	3.0	4.4	3.5	2.3	2.5	2.8	0.75
Chlorpromazine, %	2.3	1.5	2.4	3.2	3.0	1.9	2.5	2.3	0.82
Blonanserin, %	2.9	3.6	4.3	3.0	2.3	1.7	2.0	1.6	0.01
Asenapine, %	0.0	0.5	1.1	0.7	0.9	1.0	0.3	0.8	0.45
Paliperidone, %	1.0	1.5	0.5	0.5	0.0	0.8	0.5	0.3	0.04
Haloperidol, %	2.1	2.6	2.4	2.2	2.1	1.7	0.5	0.3	< 0.001
Prochlorperazine, %	0.5	0.0	0.0	0.5	0.9	0.2	0.3	0.3	0.82
Zotepine, %	1.3	0.5	0.3	0.7	0.7	0.2	0.2	0.2	0.01
Pipamperone, %	0.0	0.0	0.0	0.0	0.0	0.2	0.0	0.2	0.21
Propericiazine, %	0.5	0.8	0.3	0.0	0.0	0.0	0.3	0.2	0.10
Oxypertine, %	0.3	0.0	0.0	0.0	0.0	0.0	0.0	0.0	0.09
Clozapine, %	0.0	0.5	0.3	0.2	0.0	0.0	0.0	0.0	0.06
Fluphenazine, %	0.3	0.3	0.3	0.0	0.0	0.0	0.0	0.0	0.03
Bromperidol, %	0.3	0.0	0.0	0.0	0.0	0.0	0.0	0.0	0.09

Each drug is listed in the order of the proportion of prescriptions for antipsychotics in 2022

The Cochran–Armitage trend test was used for statistical analysis

*P* < 0.05 was considered statistically significant

## Discussion

Our results show that the prevalence of schizophrenia increased with age in males and females aged ≤ 18 years. Pediatric schizophrenia is diagnosed through clinical assessment using the American Psychiatric Association’s Diagnostic and Statistical Manual of Mental Disorders, Fifth Edition [14]. Two or more of the following characteristic symptoms must be present for a significant duration over one month (or less if successfully treated): delusions, hallucinations, disorganized speech, grossly disorganized or catatonic behavior, and negative symptoms. Therefore, patients must be old enough to communicate for an accurate diagnosis, and careful consideration is necessary regarding the prevalence observed in the 0–4-year age group in this study.

Aripiprazole and risperidone, FDA-approved for pediatric schizophrenia, were the main drugs prescribed for schizophrenia in children. Although these drugs have been tested for treating pediatric schizophrenia in RCTs [6, 8], in Japan, they remain unapproved for pediatric schizophrenia and are only approved for treating autism spectrum disorder (ASD)-associated irritability in children. Rapoport et al. reported that childhood-onset schizophrenia was usually preceded by and comorbid with ASD/pervasive developmental disorder in 30–50% of cases [15]. Although aripiprazole and risperidone are unapproved for schizophrenia in Japan, they may be prescribed for pediatric schizophrenia because it is comorbid with ASDs. Moreover, their approval for treating schizophrenia must also be considered in Japan.

The proportion of prescriptions for aripiprazole increased significantly from 2015 to 2022, whereas that for risperidone decreased significantly during the same period. Kumar et al. reported the short-term treatment efficacy and side-effect profiles of aripiprazole and risperidone for schizophrenia [16]. They found similar efficacy, while adverse events were more frequent with risperidone than with aripiprazole when assessed using the Udvalg for Kliniske Undersogelser side effect rating scale. Similarly, Coleman et al. reported that aripiprazole was superior to risperidone, considering its adverse effects [17]. Another study reported that risperidone induced more hyperprolactinemia side effects than did aripiprazole [18, 19]. Hyperprolactinemia is associated with menstrual disturbance in women [20]. Therefore, concerns about risperidone-induced hyperprolactinemia may have contributed to the marked reduction in the proportion of prescriptions for risperidone among females aged 13–18 years. Because a previous study has demonstrated similar efficacy between aripiprazole and risperidone [16], one reason for the increased trend toward aripiprazole and the decreased trend toward risperidone could be safety considerations.

We found that the prescription rates of aripiprazole and risperidone were higher in patients with schizophrenia, regardless of age, sex, and developmental disability. Aripiprazole and risperidone may be prescribed for reasons beyond efficacy and safety owing to their availability in several dosage forms, including tablets, orally disintegrating tablets, liquids, and powders. Therefore, a liquid or powder formulation can be administered to children, enabling a fine-tuned formulation design. Switching to tablets is also possible as patients age. These factors may have contributed to higher proportions of prescriptions for risperidone and aripiprazole than for other drugs.

The proportion of blonanserin prescriptions was low in our study. However, blonanserin was only approved for treating pediatric schizophrenia in Japan in 2021, resulting in the inadequate assessment of the drug within our study period; therefore, the prescribing trends must be monitored in the future.

This study has some limitations. First, regarding patients with both schizophrenia and developmental disorders, whether aripiprazole and risperidone were prescribed off-label specifically for schizophrenia or both schizophrenia and comorbid ASD is unclear. Second, comparing the proportion of antipsychotic prescriptions with that of each antipsychotic prescription may reveal concomitant cases. However, the MDV analyzer cannot distinguish concomitant from switched cases when multiple medications are prescribed within the same period. Third, the MDV analyzer does not provide information on the prescribing practices of general practitioners. Finally, this study was limited to outpatients, and patients with severe symptoms requiring hospitalization might have been excluded, possibly introducing a selection bias. Nevertheless, to our knowledge, this is the first study to clarify antipsychotic prescription trends for children in Japan. Therefore, our findings may contribute to the standardization of antipsychotic treatments for pediatric schizophrenia.

## Conclusions

Aripiprazole and risperidone were primarily prescribed for pediatric schizophrenia in Japan during the study period. Additionally, the proportion of aripiprazole prescriptions increased over time.

## Data Availability

Not applicable.

## Abbreviations

ASD: Autism spectrum disorder
DPC: Diagnosis procedure combination
FDA: Food and Drug Administration
RCT: Randomized clinical trial
